# B-mode ultrasound and dynamic contrast-enhanced ultrasound (DCE-US) of the bowel wall in patients with gastrointestinal food allergy in comparison to Crohn's disease and healthy controls

**DOI:** 10.1055/a-2735-9906

**Published:** 2025-12-12

**Authors:** Dane Wildner, Martin Raithel, Maximilian J Waldner, Alexander F. Hagel, Markus F Neurath, Lukas Pfeifer

**Affiliations:** 1 9171 Department of Internal Medicine 1, Friedrich-Alexander-Universität Erlangen-Nürnberg, Erlangen, Germany

**Keywords:** food allergy, Crohn’s disease, DCE-US, bowel ultrasound, dynamic contrast-enhanced ultrasound

## Abstract

**Purpose:**

The aim of this study was to assess bowel wall thickness using B-mode ultrasound (US) and perfusion measurement by DCE-US in patients with a food allergy (FA) compared to healthy controls (HCs) and patients with active Crohn's disease (CD).

**Materials and Methods:**

Bowel wall thickness and perfusion were assessed in FA patients as well as in HCs on a potato rice diet (PRD) and after a provocation diet (PD). Additionally, patients with active CD were examined for further comparison.

**Results:**

A total of 48 individuals (20 with FAs, 20 with CD, and 8 HCs) were included. There was no significant difference between the HCs and patients with FAs regarding the thickness of the terminal ileum (1.8mm vs. 2.2mm; p=0.46) and the sigmoid colon (2.1mm vs. 2.1mm; p=1) on a PRD. After a PD, the median value was significantly lower in the terminal ileum for HCs compared to patients with FAs (1.6 mm vs. 2.3 mm; p=0.03). In CD patients, the thickness of the terminal ileum was far more pronounced (median thickness 6 mm) compared to HCs and patients with FAs (p<0.001). There was no statistically significant difference for all tested DCE-US parameters in the terminal ileum between the HCs and patients with FAs on either PRD or PD. However, DCE-US perfusion parameters (PE, WiAUC, WiR, WiPi, and WoR) were significantly higher in patients with CD compared to HCs and patients with FAs on a PD.

**Conclusion:**

Assessment of wall thickening of the terminal ileum using US and perfusion measurements via DCE-US appears to be insufficient for distinguishing between HCs and patients with FAs. However, US and DCE-US could be helpful in differentiating patients with CD from those with FAs.

## Introduction

Food allergies have evolved into a significant health issue worldwide in the last few decades. The prevalence of self-reported food allergies is high at approximately around 17% 1 2 . The number of patients with a convincing clinical history or positive food challenge is currently low, affecting around 3–5% of the adult population in double-blind food challenge tests and up to 8% among children 3 4 5 6 .

Gastrointestinal symptoms such as abdominal pain and diarrhea are frequently encountered in cases of food allergy. These recurrent symptoms frequently overlap with those of inflammatory bowel disease (IBD) 3 7 8 . In IBD, B-mode ultrasound is well established and wall thickening is a key feature for assessing disease activity 9 . Additionally, ultrasound contrast agents have demonstrated high sensitivity for the detection of perfusion. A strong correlation between contrast enhancement in the bowel wall and inflammatory activity in Crohn’s disease has been shown. Different parameters of perfusion kinetics determined by dynamic contrast-enhanced ultrasound (DCE-US) of the bowel and correlating with disease activity were identified 10 11 12 . Ultrasound and contrast-enhanced ultrasound are readily accessible and have an excellent safety profile 13 . However, there is only limited data about B-mode ultrasound and, to the best of our knowledge, no data about DCE-US in the evaluation of patients with gastrointestinal food allergies 14 .

Considering that diagnosing food allergies remains challenging and noninvasive tests are still being explored, we designed this pilot study to assess bowel thickness using B-mode ultrasound and to measure perfusion by means of DCE-US in patients with food allergies. The results are compared to those of healthy controls and patients with active Crohn’s disease.

## Materials and Methods

### Patients

Patients with a known gastrointestinal food allergy (FA) or active ileal Crohn’s disease (CD) scheduled for therapy with anti-TNFα antibodies were recruited. Healthy, food-tolerant volunteers without any abdominal complaints served as healthy controls (HC).

## Inclusion criteria

### Patients with a food allergy

All patients with an FA were in a clinically stable condition. The FA had to have been confirmed previously by an extensive workup as either an IgE or non-IgE-mediated gastrointestinal food allergy. The prior diagnostic evaluation included anamnesis, H2 breath tests, the exclusion of celiac disease, performance of prick tests, determination of blood IgE levels as well as intestinal IgE level measurement by endoscopically guided segmental gut lavage to determine intestinal TNFα, specific and total intestinal IgE levels 15 16 .

An FA was diagnosed by either double-blind or single-blind food challenge tests with placebo controls 16 17 . Only patients meeting the appropriate laboratory criteria during allergen provocation tests and with reproducible clinical reactions during the food challenge procedures were included. All clinical symptoms were graded using the Erlangen symptom score for FAs, a standardized scoring system for gastrointestinal-mediated allergies 17 . All controls reported no clinical symptoms after food consumption and did not suffer from other allergic diseases, such as rhinitis.

### Patients with Crohn’s disease

Only treatment-naïve patients with endoscopically and histologically confirmed Crohn's disease who were scheduled for anti-TNFα therapy were included in the study. The clinical disease activity was classified using the Harvey-Bradshaw Index (HBI).

### Exclusion criteria

Pregnant women, breastfeeding mothers, patients under 18, and patients with contraindications for the use of the ultrasound contrast agent were not included in the study. Patients with a confirmed FA and persistent abdominal symptoms despite allergen avoidance were excluded as well. Patients in the CD group who had received any IBD-related medication were excluded, as previous research suggests that CEUS parameters are influenced by pharmacologic therapy, even before changes become visible in other ultrasound activity parameters 18 .

### Study design

Initially, all ultrasound examinations in the HC and FA groups were performed after an overnight fast, followed by a breakfast consisting of a hypoallergenic diet composed of non-sparkling water and a Potato Rice Diet (PRD). At a subsequent appointment, a provocation diet (PD) was administered, once again after an overnight fast. Patients with an FA consumed approximately 10–20 grams of their previously diagnosed food allergen (e. g., wheat, egg, milk). Clinical reactions were recorded and semiquantitatively assessed using the symptom score 17 .

For comparison, HC participants consumed food with a high level of histamine (salami, tuna crème, chocolate, matured cheese, coffee, sparkling wine). Ultrasound examinations were conducted at least three hours after the last food intake. CD patients were examined without any food restrictions, following consumption of a balanced diet.

### Ethical statement

The study was performed in accordance with the guidelines for Good Clinical Practice [E6(R2)] and the ethical guidelines of the Declaration of Helsinki. The study was approved by the institutional ethics committee and registered at ClinicalTrials.gov (Identifier: NCT05768971). Informed written consent was obtained from all patients and healthy volunteers.

### B-mode ultrasound

**Fig. 1:**
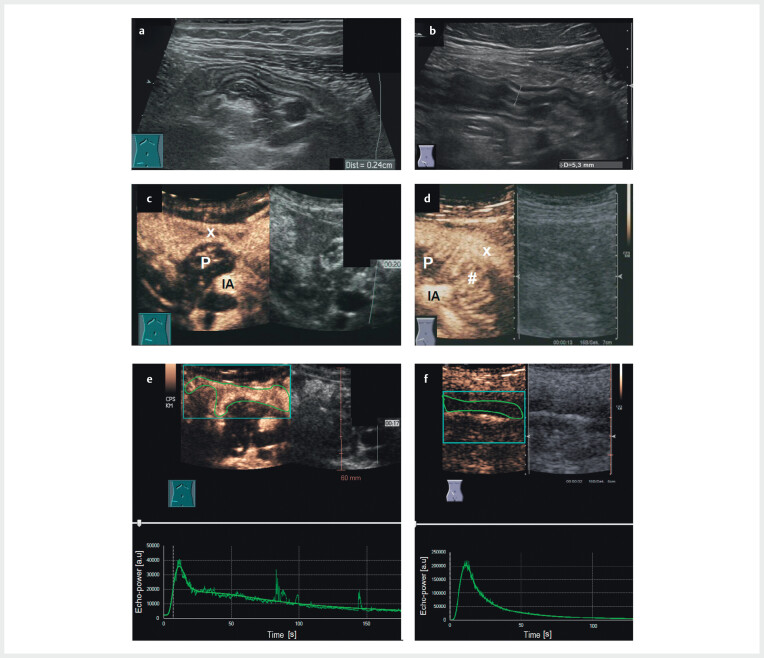
Left column: Patient with a food allergy (FA). Bowel wall thickness on B-mode ultrasound ( **a** ), perfusion of the terminal Ileum on CEUS ( **c** ), and perfusion quantification of the terminal ileum with DCE-US ( **e** ). Right column: Patient with active Crohnʼs disease (CD). Bowel wall thickness on B-mode ultrasound ( **b** ), perfusion of the terminal Ileum on CEUS ( **d** ), and perfusion quantification of the terminal ileum with DCE-US ( **f** ). x: Terminal Ileum on CEUS; P: musculus psoas major; IA: iliac artery; #: “comb sign” – referring to the active mesenteric inflammation in Crohn's disease outlined by the fibrofatty proliferation of the mesenteric tissue with pronounced intestinal blood vessels.

An experienced sonographer, who was not blinded to the different groups in this pilot study, carried out the ultrasound investigations. For the abdominal ultrasound examination of the HCs and FA patients, a Siemens Acuson Sequoia 512 was used. For CD patients, a Siemens Acuson S2000 ultrasound system (Siemens Medical Solution, Erlangen, Germany) was used. Wall thickness was assessed with a high-frequency probe (9L4 or 17L5HD) in healthy controls as well as in patients with a food allergy ( Fig. 1a )) in the terminal ileum and sigmoid colon on a PRD and a PD. The bowel wall thickness of the terminal ileum was also measured in patients with Crohn's disease ( Fig. 1b )).

### Dynamic contrast-enhanced ultrasound (DCE-US)



**Video 1** Case presentation of patients with a food allergy and active Crohn's disease on B-mode ultrasound, CE-US, and DCE-US.


Contrast-enhanced ultrasound (CEUS) examinations were performed with the same ultrasound machines using a specific CEUS preset (Cadence-Contrast Pulse Sequencing – CPS) in a dual window mode and with the corresponding convex probes (4C1 or 6C1HD). After placement of the ultrasound probe in the projection area of the terminal Ileum, a bolus injection of the ultrasound contrast agent (UCA) SonoVue (Bracco S.P.A, Milano, Italy) was performed. An assistant administered 2.4 mL of UCA followed by a 10mL flush of saline solution through a peripheral vein catheter. The raw data of the CEUS examination were recorded continuously in DICOM format for at least two minutes ( Fig. 1c , Fig. 1d , and **Video 1** ). Quantification was performed with the VueBox software (Bracco Suisse SA – Software Applications, Genève – Suisse) as shown in Fig. 1e (FA) and Fig.1f (CD). The details of the ultrasound devices, probes, and settings were entered into the software’s input form. This ensures comparability of measurements, regardless of the devices and settings being used. In the video clip, a region of interest was then selected within a consistently reproducible bowel segment of the terminal ileum and sigmoid colon. The following DCE-US parameters were calculated: Peak enhancement (PE), wash-in area under the curve (WiAUC), rise time (RT), mean transit time local (MTT), time-to-peak (TTP), wash-in rate (WiR), wash-in perfusion index (WiPI), wash-out area under the curve (WoAUC), wash-in wash-out area under the curve (WiWoAUC), fall time (FT), and wash-out rate (WoR).

### Statistical analysis

For statistical analysis, GraphPad Prism (Version 5, GraphPad Software Inc., La Jolla, USA) was used. The two-tailed Mann-Whitney test was applied for comparing wall thickness and DCE-US parameters. Tests were considered statistically significant for p < 0.05. Unless otherwise stated, the median is shown with the 25% und 75% percentiles in square brackets.

## Results

### Patients

A total of 20 patients with FAs, 20 patients with CD, and 8 HCs were included in the study. The median age was 46 years for FA patients, 47 years for CD patients, and 31 years for the HCs. The male-to-female ratio was 11:9 for FA patients, 11:9 for CD patients, and 4:4 for HCs ( Table 1 ). CD patients had a median Harvey-Bradshaw Index of 10 points, ranging from 3 to 31 points. The type of allergy and symptom presentation are displayed in Table 2 . The median increase in Food Allergy Symptom Score was 5 points, ranging from 2 to 12 points.

**Table:** **Table 1** Patient characteristics.

	Total	Food allergy	Crohn’s disease	Healthy controls
Number of individuals (n)	48	20	20	8
Median age in years (range)	45 (20–69)	46 (25–69)	47 (20–69)	31 (25–50)
Male gender (%)	26 (54)	11 (55)	11(55)	4 (50)
Median increase of Food Allergy Symptom Score in points (range)	–	5 (2–12)	–	–
Median Harvey-Bradshaw Index (HBI) (range)	–	–	10 (3–31)	–

**Table:** **Table 2** Characteristics and number of patients with a food allergy.

Type of allergy	
Type I – Local IgE	9
Type I – Systemic IgE	5
Type I – Combined local and systemic IgE	5
Type IV – Non-IgE	1
Symptoms after provocation diet (multiple choices allowed)	
Diarrhea	11
Abdominal pain	9
Cardio-pulmonary symptoms	6
Skin reactions (flush/pruritus)	4
Bloating	3
Nausea/vomiting	2
Allergen (multiple choices allowed)	
Nuts	6
Wheat	5
Egg	4
Pork	3
Beef	2
Soy	1
Rye	1

### Bowel wall thickness

**Fig. 2:**
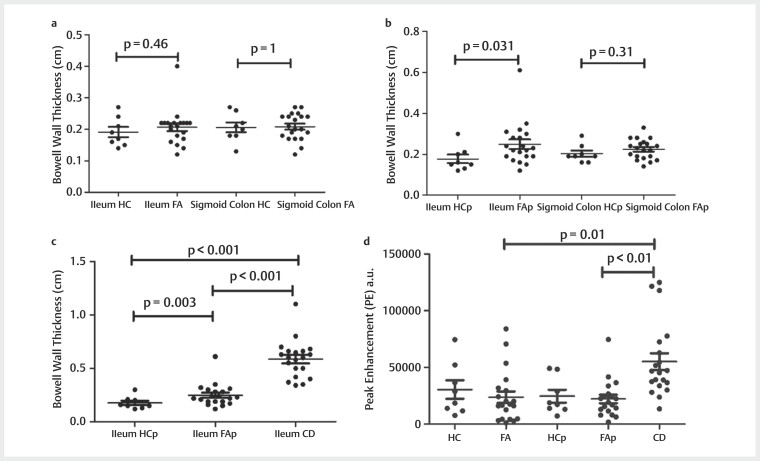
Differences in bowel wall thickness (BWT) on B-mode ultrasound and perfusion assessed by the DCEUS parameter peak enhancement (PE). **a** ) BWT of the terminal ileum and sigmoid colon in patients with a food allergy and healthy controls on a mild diet (PRD); **b** ) BWT of the terminal ileum and sigmoid colon in patients with a food allergy and healthy controls after a provocation diet (PD); **c** ) BWT of the terminal ileum in patients with a food allergy and healthy controls after a PD and patients with active Crohn's disease; **d** ) Bowel wall perfusion displayed by PE in patients with a food allergy and healthy controls after a PRD and a PD compared to patients with active Crohn's disease.

At initial presentation on a hypoallergenic diet, there was no significant difference between the HCs and FA patients with regard to the bowel wall thickness of the terminal ileum (1.8mm [0.15; 0.23] vs. 2.2mm [0.17; 0.22]; p=0.46) and the sigmoid colon (2.1mm [0.18; 0.25] vs. 2.1mm [0.17; 0.24]; p=1), as shown in Fig. 2a . After consumption of a PD, the median bowel wall thickness in the terminal ileum was significantly lower in the HCs compared to the FA patients (1.6 mm [0.14; 0.21] vs. 2.3 mm [0.19; 0.30]; p=0.03; AUROC 0.77), without a significant change in the sigmoid colon: HCp (HCs after PD: 1.9 mm [0.17; 0.23] vs. FAp (FA patients after PD: 2.3 mm [0.18; 0.26]; p=0.31), as outlined in Fig. 2b . The median bowel wall thickness of the terminal ileum in active Crohn’s disease was 6 mm [4.40; 6.60], which is significantly greater than in the HC group (p<0.01; AUROC 1) and the FA group (p<0.01; AUROC 0.97), as shown in Fig.2c .

### Bowel wall perfusion

There was no statistically significant difference in any of the tested DCE-US parameters for the terminal ileum between the HCs and the FA patients, regardless of whether patients were on a PRD or PD ( Table 3 ). For the following DCE-US parameters, there was a significant difference between the HCs on a PD and the FA patients on a PD compared to the CD patients: PE, WiAUC, WiR, WiPi, WoR ( Table 3 and Fig. 2d ). The PE for the different groups with corresponding p-values is shown. Quantitatively, the PE was twice as high in CD patients compared to the HCp group (p=0.01) and FAp group (p<0.0001).

**Table:** **Table 3** DCE–US parameters of patients with a food allergy (FA) after a provocation diet (PD) compared to healthy controls (HCs) and patients with Crohn’s disease (CD).

DCE-US parameter	Patient group			p-values		
	HC-PD	FA-PD	CD	HC-PD vs. CD	FA-PD vs. CD	HC-PD vs. FA-PD
PE	18103 (13336, 43569)	19246 (11528, 26710)	46051 (34504, 70125)	0.01	<0.01	0.73
WiAUC	129385 (114637, 217795)	123284 (79987, 236121)	241074 (175035, 398646)	0.02	<0.01	0.77
RT	9.5 (7.5, 12.5)	10.6 (7.6, 13.8)	7.6 (7.1, 8.8)	0.35	0.35	0.35
TTP	13.7 (12.4,16.4)	15.4 (10.8, 20.2)	12.5 (11.4, 15.3)	0.21	0.35	0.98
mTT	112.5 (61.1, 211.1)	93 (51.6, 162.1)	55.1 (39.3, 87.6)	0.05	0.09	0.28
WiR	2966 (1549, 6749)	2642 (1553, 4233)	7544 (5545, 13632)	0.01	<0.01	0.73
WiPi	12012 (9010, 27354)	12554 (7146, 17163)	28646 (21714, 47373)	0.02	<0.01	0.73
WoAuc	333563 (26598, 461555)	305518 (183022, 749898)	456719 (278127, 825383)	0.20	0.11	0.98
WoR	766 (481, 2531)	573 (321, 1337)	3738 (1876, 4671)	0.02	<0.01	0.44
FT	23.0 (13.2, 34.2)	29.6 (18.3, 47.5)	15.9 (11.5, 19.1)	0.20	<0.01	0.18
WiWoAuc	452040 (396984,648293)	429201 (263010,1003000)	708315 (456679,1268000)	0.11	0.05	0.98

Values are shown **as medians** with **25-75% percentile** in brackets for healthy controls on a provocation diet (HC-PD), patients with a food allergy on a provocation diet (FA–PD), and for patients with **Crohn’s** disease (CD).

For PE, the AUROC was 0.81 for differentiating the HCs on a PD from the CD patients and 0.88 for differentiating FA patients on a PD from CD patients.

## Discussion

Abdominal symptoms suspicious for food allergy are a relevant and increasing health issue in the Western world. As a result, ultrasonographers will encounter an increasing number of patients presenting with these symptoms in their daily practice. Therefore, it is important to determine whether abdominal sonography and/or DCE-US can help clinicians to identify findings associated with allergic diseases, thereby aiding in the diagnosis of these patients.

As shown in our data, there was no significant difference in bowel wall thickness between HCs and FA patients on a hypoallergenic diet. This finding might be explained by the fact that gastrointestinal allergic reactions are specifically related to the causative allergen and are reversible when the allergen has been degraded and all abnormal mediators are abolished within a timeframe of 6–72 hours 4 . Although delayed-phase reactions have occasionally been described 19 , no delayed or chronic allergic symptoms were observed in our population at the time of the ultrasonographic examination.

A few previous studies have reported bowel wall thickening in pediatric patients with a food allergy on a provocation diet 14 20 . Epifanio et al. described wall thickening of the jejunum and ileum in children with a cow’s milk allergy. Quantitatively, this study reported an increase in small bowel wall thickness between 1 and 2 mm in the terminal ileum among children experiencing abdominal symptoms following allergen exposure. The authors concluded that differences in bowel wall thickness appear to have little clinical significance, as the difference between the control group and the FA patients was minimal 20 . Similarly, we observed a significant difference in the wall thickness of the terminal ileum in FA patients compared to HCs on both a PRD and a PD. However, no such difference was observed in the sigmoid colon. This increased wall thickening in allergic patients may arise primarily from mucosal reactions such as edema, vasodilation, capillary leakage, and cellular influx into the intestinal mucosa, as recently described using confocal endomicroscopy 21 22 . However, the change in wall thickness in our study was small and does not provide a strong clinical discrimination parameter. The observed bowel wall thickness values were all within a range generally assumed to be normal (<3mm). Thus, our finding of a statistically significant, but quantitatively minor increase in ileal wall thickness three hours after provocation does not seem to provide high diagnostic value for FA patients. Further analysis should focus on later time points, a more prolonged allergen provocation diet phase (e. g., 1–5 days) and/or more severe intestinal reactions. Our single in vivo provocation before sonographic evaluation may have been too short and future sonographic examinations may be conducted after an intensified provocation period.

Regarding food allergies, there is limited data in the literature on the use of perfusion measurement with color Doppler ultrasound. In children with a cow’s milk allergy, an increase in mesenteric and bowel wall perfusion measured by color Doppler has been reported 23 . Similarly, quantitative differences during food challenge tests were detected by the vessel density in the small intestine of children with a cow’s milk allergy 20 . However, bloody stool was a common finding in these children, which was absent in our FA patients, indicating more severe illness in those children. Generally, compared to conventional Doppler ultrasound, DCE-US has the advantage of being more sensitive and, since enhancement is quantifiable, it provides a more objective assessment 12 . In addition to bowel wall thickness, we evaluated bowel wall perfusion using DCE-US. We did not observe any difference in the tested DCE-US parameters between the HCs and FA patients on a PD. To the best of our knowledge, DCE-US has not been previously evaluated in food allergies. Our data suggest that DCE-US parameters cannot display functional changes in FAs between PDR and PD. Therefore, novel functional imaging techniques should be explored for this patient group. These may include multispectral optoacoustic tomography (MSOT), which quantifies hemoglobin levels in the intestinal wall using the photoacoustic effect. Recent studies have demonstrated the ability of MSOT to detect different grades of inflammation in Crohn’s disease 24 25 , suggesting its theoretical potential for evaluating mucosal inflammation in patients with FAs as well. Another promising technique is superb microvascular imaging (SMI), which enables high-resolution visualization of vascular perfusion 26 . However, no data are currently available on SMI in the context of mucosal inflammation. In addition to bowel wall thickness, perfusion measured by DCE-US showed good discrimination between CD patients and FA patients in our cohort. The increase in bowel wall thickness and perfusion in CD patients is in line with previously published data 11 27 .

In contrast to the HCs and FA patients, the CD patients had significantly increased bowel wall thickness at initial presentation. This reflects the known transmural inflammation characteristic of CD. The significantly thinner bowel wall seen in FA patients compared to CD patients may be partly explained by the preferential localization of allergic immune effector cells, such as mast cells and eosinophils, in the superficial mucosal layers (e. g., intraepithelial and lamina propria) whereas they are rarely found in deeper layers such as the submucosa, muscularis propria, and serosa 28 29 . Bowel wall thickening in the CD patients was substantially more pronounced than in the FA patients and the HCs, demonstrating excellent discrimination feasibility between CD patients and FA patients/HCs irrespective of food ingestion.

One limitation of our study is that the FA patients ingested approximately 10–20 g of their known allergen before sonographic examination. The allergen dose was relatively low due to concerns about potential adverse reactions during open challenge tests. However, strict diagnostic criteria were used to confirm the food allergy. Although, our findings may not be diagnostically valuable for FA patients as a whole, future studies should focus on assessing more severe individual reactions with higher allergen ingestion to evaluate bowel responses. Another potential limitation of the ultrasound global assessment is its inherent subjectivity. Future investigations should, therefore, involve multiple sonographers performing structured ultrasound examinations, including an assessment of the inter-rater agreement. Another limitation regarding the CD group is that patients were scheduled for biological therapy and, therefore, milder forms of ileal CD were not included in the study.

In summary, our study suggests that bowel wall thickness of the terminal ileum is altered in FA patients on a PD compared to HCs. Although the results were statistically significant, their clinical value is insufficient for distinguishing between HCs and FA patients. In contrast, wall thickness was notably greater in CD patients than in FA patients, indicating that wall thickness assessment may be more useful for differentiating between FAs and more pronounced inflammatory diseases, such as CD. Perfusion assessed by a dynamic contrast ultrasound-based approach (DCE-US) was much higher in CD patients but showed no significant changes in FA patients compared to HCs and may, therefore, also be especially helpful in the differentiation between FAs and other diseases like CD, regardless of diet. Future studies should explore novel imaging techniques such as multispectral optoacoustic tomography or superb microvascular imaging in FA patients.

## Notes

Abbreviations **AUROC** area under the receiver operating characteristic
**CD** Crohn’s Disease
**CEUS** contrast-enhanced ultrasound
**CPS** cadence-contrast pulse sequencing
**DCE-US** dynamic contrast-enhanced ultrasound
**DICOM** Digital Imaging and Communications in Medicine
**e. g.** exempli gratia
**FA** food allergy
**FAp** patients with a food allergy after a provocation diet
**FT** fall time
**
H _2_** hydrogen
**HBI** Harvey-Bradshaw Index
**HC** healthy control
**HCp** healthy controls after a provocation diet
**IBD** inflammatory bowel disease
**IgE** immunoglobulin E
**MTT** mean transit time
**MSOT** multispectral optoacoustic tomography
**PD** provocation diet
**PE** peak enhancement
**PRD** potato rice diet
**RT** rise time
**TNFα** tumor necrosis factor alpha
**TTP** time-to-peak
**UCA** ultrasound contrast agent
**US** ultrasound
**SMI** superb microvascular imaging
**WiAUC** wash-in area under the curve
**WiPI** wash-in perfusion index
**WiR** wash-in rate
**WiWoAUC** wash-in wash-out area under the curve
**WoAUC** wash-out area under the curve
**WoR** wash-out rate
